# Association of Glioblastoma Multiforme Stem Cell Characteristics, Differentiation, and Microglia Marker Genes with Patient Survival

**DOI:** 10.1155/2018/9628289

**Published:** 2018-01-17

**Authors:** Sandra Bien-Möller, Ellen Balz, Susann Herzog, Laura Plantera, Silke Vogelgesang, Kerstin Weitmann, Carolin Seifert, Matthias A. Fink, Sascha Marx, Angela Bialke, Chitra Venugopal, Sheila K. Singh, Wolfgang Hoffmann, Bernhard H. Rauch, Henry W. S. Schroeder

**Affiliations:** ^1^Department of Pharmacology, University Medicine Greifswald, Greifswald, Germany; ^2^Department of Neurosurgery, University Medicine Greifswald, Greifswald, Germany; ^3^Department of Neuropathology, Institute of Pathology, University Medicine Greifswald, Greifswald, Germany; ^4^Institute for Community Medicine, University Medicine Greifswald, Greifswald, Germany; ^5^Trusted Third Party, University Medicine Greifswald, Greifswald, Germany; ^6^McMaster University Hamilton, McMaster Stem Cell and Cancer Research Institute, Hamilton, ON, Canada

## Abstract

Patients with glioblastoma multiforme (GBM) are at high risk to develop a relapse despite multimodal therapy. Assumedly, glioma stem cells (GSCs) are responsible for treatment resistance of GBM. Identification of specific GSC markers may help to develop targeted therapies. Here, we performed expression analyses of stem cell (ABCG2, CD44, CD95, CD133, ELF4, Nanog, and Nestin) as well as differentiation and microglia markers (GFAP, Iba1, and Sparc) in GBM compared to nonmalignant brain. Furthermore, the role of these proteins for patient survival and their expression in LN18 stem-like neurospheres was analyzed. At mRNA level, ABCG2 and CD95 were reduced, GFAP was unchanged; all other investigated markers were increased in GBM. At protein level, CD44, ELF4, Nanog, Nestin, and Sparc were elevated in GBM, but only CD133 and Nestin were strongly associated with survival time. In addition, ABCG2 and GFAP expression was decreased in LN18 neurospheres whereas CD44, CD95, CD133, ELF4, Nanog, Nestin, and Sparc were upregulated. Altogether only CD133 and Nestin were associated with survival rates. This raises concerns regarding the suitability of the other target structures as prognostic markers, but makes both CD133 and Nestin candidates for GBM therapy. Nevertheless, a search for more specific marker proteins is urgently needed.

## 1. Introduction

Glioblastoma multiforme (GBM) represents the most common and most aggressive primary brain tumor in adults with an almost certainly lethal outcome. Despite a multimodal therapy including surgical removal of the tumor and a radiochemotherapy, the overall survival time is still only 12 to 15 months [1, 2]. The current pathophysiological hypothesis involves so-called glioma stem cells (GSCs) being responsible for the formation, expansion, recurrence, and the high-therapy resistance of GBM [3]. The first proof of the existence of GSCs in brain tumors was reported by Singh and colleagues [4]. GSCs are CD133 positive and have the potential for self-renewal, proliferation, and differentiation [5, 6]. In a xenograft model, it was shown that implantation of CD133-positive glioma cells results in the development of intracranial tumors [5] and that these cells are resistant to radiation as well as temozolomide as the standard chemotherapeutic compound in GBM therapy [7, 8]. Furthermore, it is known that GSC growth depends on brain microenvironment including nontumorigenic cell types such as microglia and endothelial cells, which are able to influence GSC phenotype transition from precursor to differentiated cells and vice versa [9, 10].

Besides CD133, several other proteins are discussed as potential markers for GSCs including the transcription factors ELF4 [11] and Nanog [12], the transmembrane receptor CD44 [13], the efflux transporter ABCG2 [14], and the filament protein Nestin [15]. Some studies have shown expression of CD133, CD44, and ABCG2 to be negatively correlated with the survival time of GBM patients [13, 16–18].

Further, the relevance of neural progenitor cells in the context of the development of brain tumors is to date controversially discussed. Neural stem cells were identified in GBM tissue and are capable to generate these highly aggressive brain tumors [19]. Contrary to this, another study observed anticancerous effects of endogenous neural precursor cells in association with improved survival of GBM patients [20]. Moreover, microglia also facilitates the invasiveness of glioma cells, and thus, it was concluded that the tumor microenvironment might have a great impact on the aggressive behavior of GBM [21].

Since the GSCs are regarded as the most important target for new potential therapeutic options, the identification of marker proteins which impact the survival time of GBM patients might help to develop future targeted therapies.

This study represents a comprehensive analysis of the expression of the most discussed and most specific candidate stem cell (ABCG2, CD44, CD95, CD133, ELF4, Nanog, and Nestin) as well as differentiation and microglia markers (GFAP, Iba1, and Sparc) at the level of both mRNA and protein in primary GBM samples in comparison to nonmalignant brain specimens as well as in stem-like GBM neurospheres. Furthermore, utilizing the Kaplan-Meier and multivariable regression analyses, we evaluated the association of these marker proteins with the survival time of GBM patients.

## 2. Materials and Methods

### 2.1. Patient Specimens

Following an institutional review board-approved protocol (BB 089/08), fresh human GBM tissues were collected from 78 patients with primary GBM (50 males, 27 females) who underwent surgical removal of GBM within their therapeutic regime (study period from 15.10.2007 to 01.08.2014). The histological analyses are based on the 2007 World Health Organization (WHO) criteria for tumors of the central nervous system [22]. Overall survival time was defined as the time span from the date of diagnosis to the date of death. The detailed clinical characteristics are shown in Table 1. Beside GBM samples, eight nonneoplastic brain tissues (frontal/temporal lobes) from the Institute of Pathology/Department of Neuropathology of the University Greifswald were analyzed. These control brain specimens were obtained during routine autopsy. Tissue samples were cut and frozen at minus 80°C immediately after removal. The autopsy cases died of pneumonia, heart failure, sepsis, or carcinoma of pancreas, respectively. There were no neurological disorders. Furthermore, RNA and protein samples of two nonmalignant (one frontal and one temporal lobe) specimens were obtained from BioChain Institute Inc. (Newark, CA, USA).

### 2.2. Quantitative Real-Time PCR Analysis

For mRNA expression analysis, total RNA was isolated using PeqGold RNAPure (PeqLab, Erlangen, Germany) and reversely transcribed using the High Capacity cDNA Reverse Transcription Kit (Applied Biosystems by Life Technologies, Weiterstadt, Germany). The mRNA expression was analyzed by the following Gene Expression Assays on Demand from Applied Biosystems: ABCG2, Hs01053790_m1; CD133 (PROM1), Hs01009250_m1; CD44, Hs0107586_m1; CD95, Hs0110621_m1; ELF4, Hs01086126_m1; GFAP, Hs00909236_m1; Iba1 (AIF1), Hs00610419_m1; Nanog, Hs04260366_g1; Nestin, Hs04187831_m1; Sparc, Hs00234160_m1; and eukaryotic 18S rRNA endogenous control, 4319413E. Quantitative real-time PCR was performed in a 7900 HT Fast Real-Time PCR system from Applied Biosystems. Each mRNA level was normalized to 18S rRNA and analyzed by the ΔΔct method.

### 2.3. Western Blot Analysis

Protein extracts of patient's glioblastoma and control brain samples were prepared using the Qiagen TissueLyser II. Approximately 20–30 mg of the nitrogen-cooled tissue tumor sample was shredded for 90 seconds at a frequency of 30 Hz. The resulting tissue powder was dissolved immediately in precooled lysis buffer (50 mM Tris–HCl pH 7.4, 100 mM NaCl, 0.1% Triton X-100, 5 mM EDTA containing protease/phosphatase inhibitors: 1 mM PMSF, 1 mM leupeptin, 1 mM aprotinin, and 250 *μ*g/ml sodium vanadate) and incubated on ice for 45 minutes followed by a centrifugation step at 6000 rpm to remove cell debris. The BCA Protein Assay Kit (Thermo Fisher Scientific, Rockford) was used to determine the protein concentrations in the tissue extracts. Subsequently, after denaturation in Laemmli buffer at 95°C for 5 minutes, 40 *μ*g of each sample was separated on 7.5 or 10% SDS polyacrylamide gels according to the molecular weight of the respective proteins. The tank blot system (Bio-Rad, Hempstead, UK) was used for immunoblotting of the separated proteins to Whatman® nitrocellulose membrane which was afterwards blocked in 5% FCS or skim milk in Tris-buffered saline containing 0.05% Tween 20 (TBST) for 1 hour at room temperature. The following primary antibodies were diluted in TBST and 0.05% sodium azide and incubated either for 2 hours at room temperature or overnight at 4°C: mouse anti-GAPDH (Meridian Life Science Inc., Memphis, USA), mouse anti-ABCG2 (Alexis Biochemicals, CA, USA), mouse anti-CD44 (Cell Signaling Technology, Boston, USA), rabbit anti-CD95 (Bioworld Technology, MN, USA), mouse anti-CD133 (Merck Millipore, Darmstadt, Germany; Novus Biologicals, Cambridge, UK), rabbit anti-ELF4 (Abgent Inc., San Diego, USA), mouse anti-GFAP (Cell Signaling Technology, Boston, USA), goat anti-Iba1 (Novus Biologicals, Littleton, CO, USA), rabbit anti-Nanog (Cell Signaling Technology, Boston, USA) goat anti-Nestin (Santa Cruz Biotechnology Inc., Heidelberg, Germany), and rabbit anti-Sparc (Cell Signaling Technology, Boston, USA).

The secondary horseradish peroxidase-conjugated goat-anti-rabbit, goat-anti-mouse, or horse-anti-goat IgG antibodies (Vector Laboratories, Burlingame, CA) were used at a 1 : 2000 dilution for 1.5 hours at room temperature. Chemiluminescence signals were detected with ChemiDoc™ XRS Imaging System (Bio-Rad, Hempstead, UK) using ECL Plus Western Blotting Substrate (Thermo Scientific, Rockford, USA) followed by densitometric analysis (Quantity One, Bio-Rad). The relative optical densities of the specific bands were calculated and normalized to GAPDH as a loading control. Afterwards, the control brain samples were averaged and set to 1. GBM samples were related to this control value and all data were then used for the creation of box plots which represent the median as well as the 5th and 95th percentiles.

### 2.4. Cell Culture of LN18 and Primary GBM Cells

*In vitro* experiments were performed with the human LN18 GBM cell line which was obtained from the American Type Culture Collection (ATCC, Manassas, VA, USA). LN18 cells were maintained in DMEM supplemented with 10% FCS, 2 mM glutamine, and 2 mM nonessential amino acids (all from PAA Laboratories, Cölbe, Germany) at 37°C, 95% humidity, and 5% CO_2_. LN18 glioma neurospheres, which are thought to be enriched in cancer stem cells [7], are cultured with the NeuroCult™ NS-A Proliferation Kit (Human, STEMCELL Technologies, Cologne, Germany) and added with 20 ng/ml rh EGF (Firma), 10 ng/ml rh bFGF (Firma), and 0.0002% heparin (Firma) according to the manufacturer's protocol. Gene expression in LN18 neurospheres was determined in passage 2 after seeding in NeuroCult NS-A Proliferation media in comparison to the adherent cells of the same passage number cultured in DMEM media.

Further, for immunoblotting, protein pellets from the human glioma cell line U87-MG (cultured under standard conditions in MEM medium supplemented with 10% FCS, 2 mM glutamine, and 2 mM nonessential amino acids) and the human pluripotent embryonal carcinoma cell line NTERA-2 (cultured under standard conditions in DMEM medium supplemented with 10% FCS, 2 mM glutamine, and 2 mM nonessential amino acids) were used. Both cell lines were purchased from ATCC (ATCC, Manassas, VA, USA).

Primary human GBM cells (pGC) from six different tissue samples were isolated with the Brain Tumor Dissociation Kit (Miltenyi Biotec GmbH, Bergisch Gladbach, Germany) according to the manufacturer's instruction. Adherent primary tumor cells as well as A172, GaMG, and HF66 GBM cell lines were maintained in culture as described above for LN18 cells and analyzed for protein expression after two to four passages.

### 2.5. Statistical Analysis

GraphPad Prism 5.0. (GraphPad Software Inc., California, USA) was used for statistical analyses. Data of *in vitro* analyses represent 3 or 4 independent experiments (indicated in the figure legends and shown as mean ± SD). Box plots of data of patients' samples are shown as the median and the 5th and 95th percentiles. Pairwise comparisons were performed using the Mann–Whitney *U* test. The duration of a patient's overall survival (OS) was defined as the time from the first tumor detection until death. Information on vital status and date of death was obtained from the official population registry. Based on the gene expression, GBM specimens were divided into the lower half versus the upper half of gene expression level as determined by real-time PCR or immunoblot analysis (≤median versus >median expression). These data were used for calculation of hazard ratios and creation of the Kaplan-Meier graphs to investigate the association between expression status and survival time of GBM patients. For comparison of survival curves, we used both the Mantel-Cox log-rank test and the Gehan-Breslow-Wilcoxon test using GraphPad Prism. Correlations between the expression of the investigated genes or proteins and the age at diagnosis were analyzed by Spearman's nonparametric correlation. Specimens with expression rates of the target gene and the housekeeping gene lower than the detection limit of quantitative real-time PCR or Western blot were excluded from data analysis. Multivariable Cox regression analysis was performed using the statistical program STATA (Intercooled Stata/SE 11.2, StataCorp LP, Texas, USA) and adjusted for sex, age at diagnosis, and therapy regime. Levels of statistical significance were defined as ^∗^ *p* < 0.05, ^∗∗^ *p* < 0.01, and ^∗∗∗^ *p* < 0.001.

## 3. Results

### 3.1. mRNA Expression of Candidate Stem Cell Markers in GBM and Their Association with Patients' Survival

**Figure 1:**
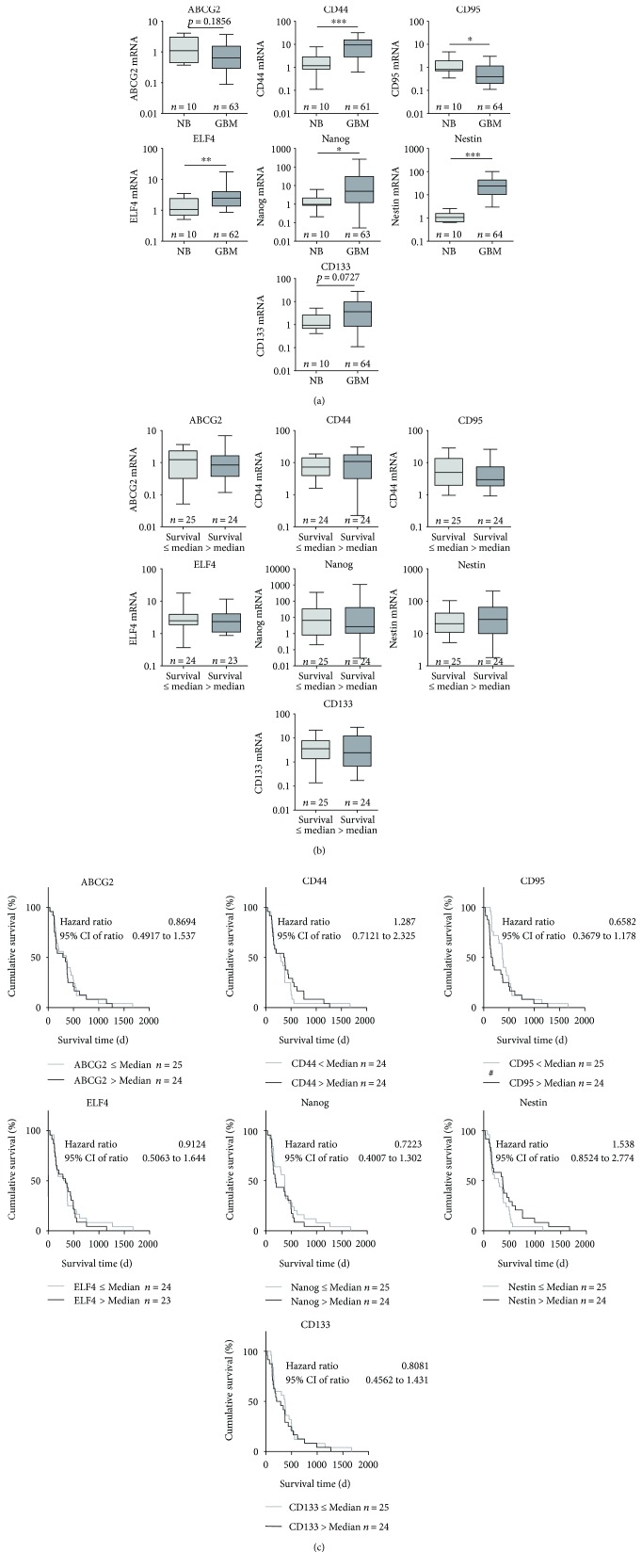
mRNA expression of the potential stem cell markers ABCG2, CD44, CD95, CD133, ELF4, Nanog, and Nestin in glioblastoma tissue. (a) Relative mRNA expression in glioblastoma (GBM) in comparison to nonmalignant brain tissue (NB) analyzed by quantitative RT-PCR with normalization to 18S rRNA in relation to the median of data is shown as box plots representing the median as horizontal bars as well as the 5th and 95th percentiles. Mann–Whitney *U* test, ^∗^ *p* < 0.05, ^∗∗^ *p* < 0.005, and ^∗∗∗^ *p* < 0.001. (b) Investigation of an impact of mRNA expression on patients' survival by subdividing our patient cohort into two groups in dependence of their survival time (≤median versus >median). Data are shown as box plots representing the median as horizontal bars as well as the 5th and 95th percentiles. Mann–Whitney *U* test, ^∗^ *p* < 0.05. (c) Kaplan-Meier survival curves for patients with GBM based on their ABCG2, CD44, CD95, CD133, ELF4, Nanog, or Nestin mRNA expression. Patients were divided into two subgroups depending on the respective median gene expression as determined by quantitative RT-PCR. Calculation of hazard ratios (≤median versus >median expression); Gehan-Breslow-Wilcoxon test, ^#^ *p* < 0.05; log-rank Mantel-Cox test, no significant differences.

Expression of candidate stem cell and differentiation markers in GBM specimens was analyzed in comparison to nonmalignant brain by quantitative RT-PCR. As seen in Figure 1(a), with the exception of ABCG2 and CD95, the mRNA of all other investigated stem cell markers was upregulated in GBM compared to healthy brain tissue. ABCG2 mRNA was not significantly changed but a slight reduction was seen from 1.09 (Min–Max: 0.37–4.09) to 0.68 (Min–Max: 0.038–7.78) in GBM. The expression level of CD44 was significantly enhanced from 1.18 (Min–Max: 0.11–7.77) in nonmalignant brain to 8.89 (Min–Max: 0.09–36.37) in GBM. In contrast, CD95 mRNA was significantly reduced from 0.82 (Min–Max: 0.35–4.70) in the normal brain to 0.38 (Min–Max: 0.06–4.76) in GBM. CD133 exhibited an increased mRNA content from 0.95 (Min–Max: 0.41–5.21) to 3.57 (Min–Max: 0.04–99.55) in GBM but this failed to reach statistical significance (*p* = 0.0879). Expression of ELF4 and Nanog mRNA was also significantly elevated in GBM compared to nonmalignant brain samples from 1.06 (Min–Max: 0.51–3.52) to 2.58 (Min–Max: 0.33–33.86) and 0.98 (Min–Max: 0.21–6.24) to 5.76 (Min–Max: 0.03–1352), respectively. For Nestin mRNA, we observed the most potent increase from 1.06 (Min–Max: 0.64–2.59) in the normal brain to 23.68 (Min–Max: 1.05–247.2) in GBM (*p* < 0.0001).

To investigate whether mRNA expression of candidate stem cell markers has any impact on the survival of patients with GBM, we utilized two different approaches. First, we subdivided our patient cohort into two groups according to their survival time (≤median versus >median, Figure 1(b)). The associated mRNA analyses did not indicate any significant differences for ABCG2, CD44, CD95 CD133, ELF4, Nanog, and Nestin.

To further evaluate these findings, we next performed gene expression based on Kaplan-Meier analyses which mostly confirmed our results (Figure 1(c)) with the exception of CD95 mRNA data. Interestingly, a CD95 mRNA expression below the median was associated with a significantly prolonged survival time at least in the first 500 days (Gehan-Breslow-Wilcoxon test, *p* = 0.0185, hazard ratio: 0.6582). The median survival time for patients with a low CD95 mRNA content (≤median) was 373 days compared to 161 days for patients with a high CD95 mRNA expression (>median). Conversely, a trend towards a worse survival was seen in patients having a Nestin mRNA expression below the median (log-rank Mantel-Cox test, *p* = 0.1529, hazard ratio: 1.538). The median survival time for patients with a low Nestin mRNA content (≤median) was 289 days compared to 370 days for patients with a high Nestin mRNA expression (>median).

In multivariable regression analyses with adjustment for sex, age at diagnosis, and therapy regime, the prognostic impact of CD95 mRNA expression was confirmed (*p* = 0.025, hazard ratio: 0.4505, Supplemental table S1C). Furthermore, for CD133 (*p* = 0.038, hazard ratio: 0.4936, Supplemental table S1D) and Nestin (*p* = 0.035, hazard ratio: 2.2143, Supplemental table S1G) a significant influence on patients' survival was now found in the multivariable analyses. Results of all multivariable regression analyses of the mRNA expression data are summarized in the Supplemental tables S1A–J.

### 3.2. mRNA Expression of Candidate Differentiation and Microglia Markers in GBM and Their Association with Patients' Survival

**Figure 2:**
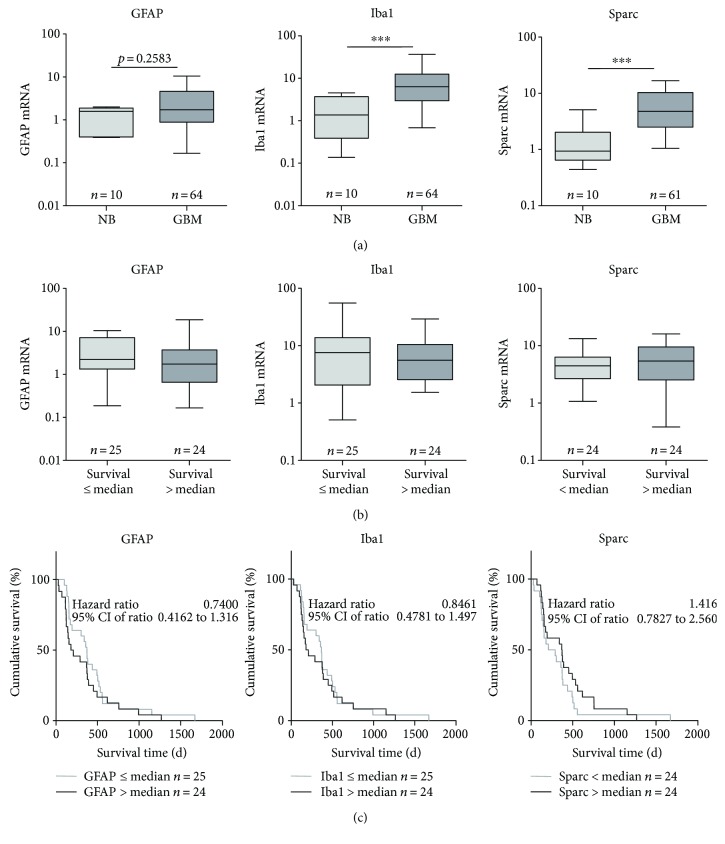
mRNA expression of candidate differentiation marker GFAP and microglia markers Iba1 and Sparc in glioblastoma tissue. (a) Relative mRNA expression in glioblastoma (GBM) in comparison to nonmalignant brain tissue (NB) analyzed by quantitative RT-PCR with normalization to 18S rRNA in relation to the median of data is shown as box plots representing the median as horizontal bars as well as the 5th and 95th percentiles. Mann–Whitney *U* test, ^∗∗∗^ *p* < 0.001. (b) Investigation of an impact of mRNA expression on patients' survival by subdividing our patient cohort into two groups in dependence of their survival time (≤median versus >median). Data are shown as box plots representing the median as horizontal bars as well as the 5th and 95th percentiles. Mann–Whitney *U* test; no significant differences. (c) Kaplan-Meier survival curves for patients with GBM based on their GFAP, Iba1, or Sparc mRNA expression. Patients were divided into two subgroups depending on the respective median gene expression as determined by quantitative RT-PCR. Calculation of hazard ratios (≤median versus >median expression); Gehan-Breslow-Wilcoxon test and log-rank Mantel-Cox test, no significant differences.

Analogous to the stem cell markers, we analyzed the mRNA expression for the astrocytic differentiation marker GFAP and the microglia markers Iba1 and Sparc (Figure 2(a)). GFAP mRNA level was not significantly different between nonmalignant control brain and GBM tissue samples (relative mRNA values of 1.58; Min–Max: 0.39–1.99 versus 1.71; Min.-Max.: 0.05–22.07). In contrast, mRNA expression of both microglia markers Iba1 and Sparc was significantly increased in GBM specimens in comparison to nonmalignant brain tissue from 1.37 (Min–Max: 0.14–4.54) to 6.53 (Min–Max: 0.27–57.73) for Iba1 and from 0.94 (Min–Max.: 0.44–5.10) to 4.82 (Min–Max: 0.28–35.97) for Sparc.

Subdividing of the GBM patient cohort into two subgroups depending on the survival time (≤median versus >median) revealed no significant associations of mRNA levels with survival for GFAP, Iba1, or Sparc (Figure 2(b)).

The Kaplan-Meier analyses (Figure 2(c)) did also not reveal significant associations between patients' survival times and GFAP, Iba1, or Sparc mRNA expression. Only a trend towards a prolonged survival time in the first 500 days of the survival period was seen for patients with a lower GFAP mRNA expression (Gehan-Breslow-Wilcoxon test, *p* = 0.1141, hazard ratio: 0.74). The median survival time for patients with a low GFAP mRNA content (≤median) was 372 days compared to 194 days for patients with a high GFAP mRNA expression (>median).

In addition, Kaplan-Meier analyses of Sparc mRNA levels also showed a slight trend towards a prolonged survival time for patients having a higher Sparc mRNA expression but this effect was only visible after 200 days of the survival period (Gehan-Breslow-Wilcoxon Test, *p* = 0.1974, hazard ratio: 1.416). The median survival time for patients with a high Sparc mRNA content (≤median) was 370 days compared to 249 days for patients with a low Sparc mRNA expression (>median). Of note, multivariable regression analysis with adjustment for sex, age at diagnosis, and therapy regime revealed a significant prognostic influence of Sparc mRNA on patients' survival (*p* = 0.028, hazard ratio: 2.4198, Supplemental table S1I).

For Iba1 mRNA expression, the Kaplan-Meier analysis revealed no trends for an association with the survival time of patients with GBM (Figure 2(c)).

### 3.3. Protein Expression of Candidate Stem Cell Markers in GBM and Their Association with Patients' Survival

**Figure 3:**
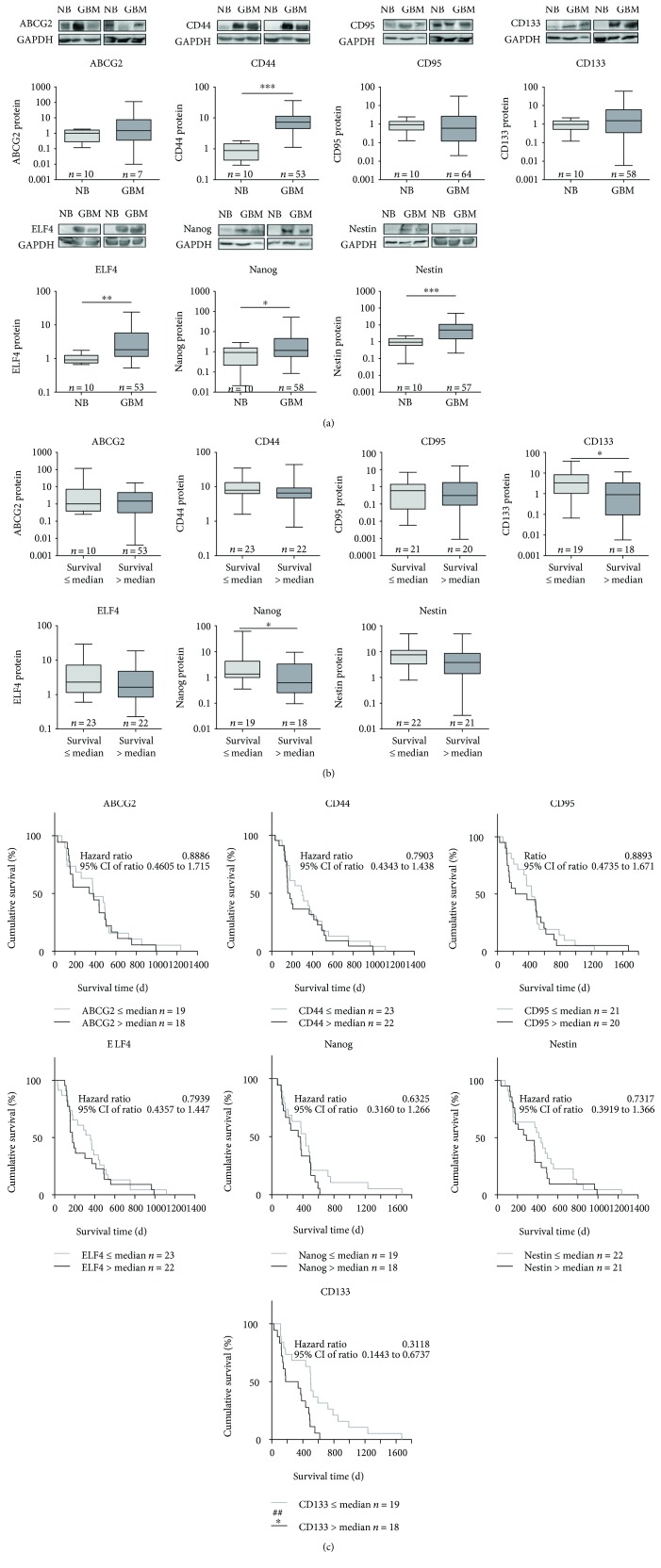
Protein expression of the potential stem cell markers ABCG2, CD44, CD95, CD133, ELF4, Nanog, and Nestin in glioblastoma tissue. (a) Protein expression in glioblastoma (GBM) in comparison to nonmalignant brain tissue (NB) determined by Western blot with densitometric analysis. The relative optical densities of the specific bands were calculated and normalized to GAPDH as a loading control in relation to the median of NB. Data are shown as box plots representing the median as horizontal bars as well as the 5th and 95th percentiles. Mann–Whitney *U* test, ^∗∗^ *p* < 0.005 and ^∗∗∗^ *p* < 0.001. (b) Investigation of an impact of marker protein expression on patients' survival by subdividing our patient cohort into two groups in dependence of their survival time (≤median versus >median). Data are shown as box plots representing the median as horizontal bars as well as the 5th and 95th percentiles. Mann–Whitney *U* test, no significant differences. (c) Kaplan-Meier survival curves for patients with GBM based on their ABCG2, CD44, CD95, CD133, ELF4, Nanog, or Nestin protein expression. Patients were divided into two subgroups depending on the respective median gene expression as determined by quantitative RT-PCR. Calculation of hazard ratios (≤median versus >median expression); Gehan-Breslow-Wilcoxon test, ^##^ *p* < 0.01; log-rank Mantel-Cox test, ^∗^ *p* < 0.05.

The protein expression of candidate stem cell markers was analyzed by immunoblotting extracts from both glioblastoma and nonmalignant brain tissue. The results are shown in Figure 3(a). For ABCG2, CD95, and CD133, the protein level in GBM tissue was nearly the same as in the healthy brain. In contrast, median protein expression of CD44, ELF4, Nanog, and Nestin was significantly increased in GBM compared to nonmalignant brain from 0.88 (Min–Max: 0.29–1.84) to 7.31 (Min–Max: 0.57–45.34) for CD44, from 0.91 (Min–Max: 0.66–1.77) to 1.84 (Min–Max: 0.19–29.28) for ELF4, from 0.93 (Min–Max: 0.02–2.93) to 1.17 (Min–Max: 0.01–199.3) for Nanog, and from 0.94 (Min–Max.: 0.05–2.29) to 5.01 (Min–Max: 0.01–295.2) for Nestin, respectively.

The median analysis with splitting of the GBM patient cohort into two subgroups depending on the survival time showed no significant differences in the protein expression of ABCG2, CD44, CD95, ELF4, and Nestin, as demonstrated in Figure 3(b). In contrast, for CD133 and Nanog, a significant difference in protein expression was seen between patients who survived longer or shorter than the median survival time. In patients with a prolonged survival time, the protein expression of CD133 and Nanog was significantly reduced from 3.29 (Min–Max: 0.07–37.92) to 0.89 (Min–Max: 0.006–11.33) and from 1.35 (Min–Max: 0.36–62.09) to 0.63 (Min–Max: 0.09–9.50), respectively.

These results were complemented by the Kaplan-Meier analyses. Expression levels of ABCG2, CD44, CD95, ELF4, and Nestin were not associated with survival times. In addition, also, Nanog protein content exhibited only a trend towards a decreased survival time (log-rank Mantel-Cox test, *p* value 0.1958, hazard ratio 0.6325, Figure 3(c)). The median survival time for patients with a high Nanog protein expression (>median) was 354.5 days in comparison to 435 days in patients with a low Nanog protein content (≤median).

Interestingly, CD133 was the only protein whose expression level was significantly associated with the survival time of GBM patients in univariate analyses. Here, a prolonged survival time was seen in patients with a low CD133 protein expression (≤median) within the tumor (hazard ratio: 0.3118). The median survival time of patients with a high CD133 protein level (>median) was 258.5 days compared to a nearly twice as long survival period of 502 days in GBM patients with a low CD133 protein expression (≤median). This prognostic impact of CD133 protein expression was also present in multivariable analyses with adjustment for sex, age at diagnosis, and therapy regime, as seen in the Supplemental table S2D (*p* = 0.017, hazard ratio: 0.3323).

Noteworthy, patients having a high Nestin protein expression (>median) showed a worse survival time (median survival 289 days) compared to patients with a lower Nestin expression (median survival 424 days). However, this was observed only in the time range between 200 and 800 days and did not reach overall statistical significance. Interestingly, in multivariable analyses with adjustment for sex, age at diagnosis, and therapy regime, Nestin protein expression was significantly associated with patients' survival time (*p* = 0.003, hazard ratio: 0.2857, Supplemental table S2G). Results of all multivariable analyses of protein expression data are summarized in the Supplemental tables S2A–I.

### 3.4. Protein Expression of Candidate Differentiation and Microglia Markers in GBM and Their Association with Patients' Survival

**Figure 4:**
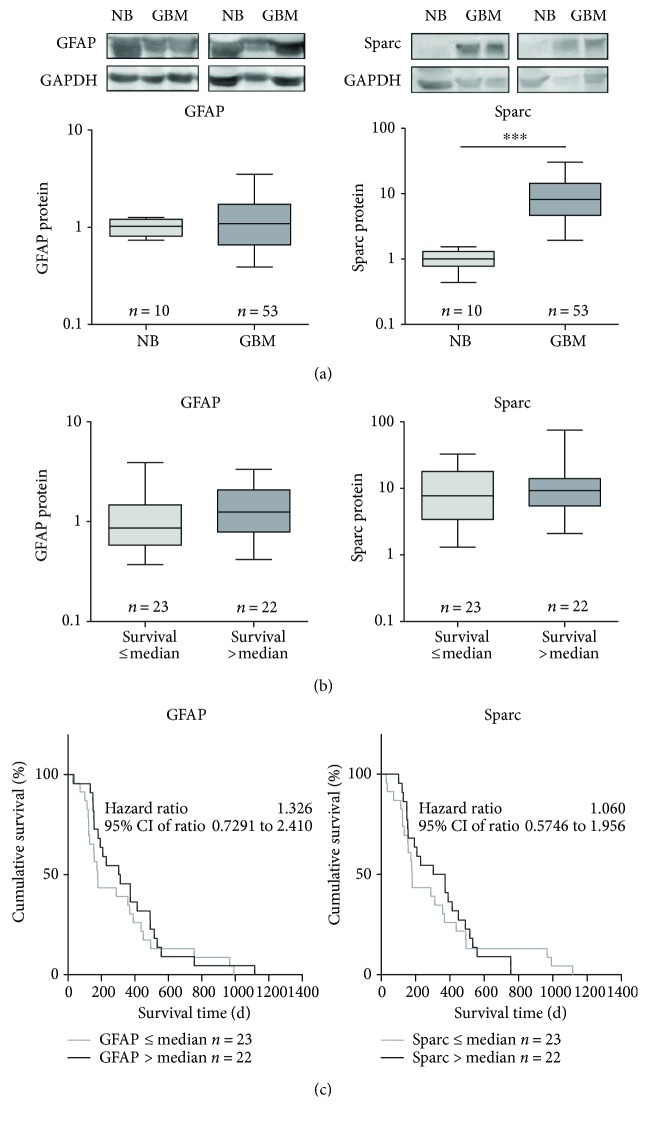
Protein expression of candidate differentiation marker GFAP and microglia markers Iba1 and Sparc in glioblastoma tissue. (a) Protein expression in glioblastoma (GBM) in comparison to nonmalignant brain tissue (NB) determined by Western blot with densitometric analysis. The relative optical densities of the specific bands were calculated and normalized to GAPDH as a loading control in relation to the median of NB. Data are shown as box plots representing the median as horizontal bars as well as the 5th and 95th percentiles. Mann–Whitney *U* test, ^∗∗∗^ *p* < 0.01. (b) Investigation of an impact of protein expression on patients' survival by subdividing our patient cohort into two groups in dependence of their survival time (≤median versus >median). Data are shown as box plots representing the median as horizontal bars as well as the 5th and 95th percentiles. Mann–Whitney *U* test; no significant differences. (c) Kaplan-Meier survival curves for patients with GBM based on their GFAP, Iba1, or Sparc protein expression. Patients were divided into two subgroups depending on the respective median gene expression as determined by quantitative RT-PCR. Calculation of hazard ratios (≤median versus >median expression); Gehan-Breslow-Wilcoxon test and log-rank Mantel-Cox test, no significant differences.

In accordance to the mRNA data, the protein content of GFAP was not significantly altered in GBM tissue compared to nonmalignant brain (relative protein values: 1.09, Min–Max: 0.37–4.04 versus 1.03, Min–Max: 0.74–1.26), while Sparc protein expression was significantly upregulated in GBM from 1.01 (Min–Max: 0.44–1.54) in control brain tissue to 8.14 (Min–Max: 1.16–82.85; Figure 4(a)). Signals for Iba1 protein—despite using three different antibodies—were neither detectable in the nonmalignant brain nor in GBM samples (not shown).

Subdividing our patient cohort into two subgroups according to their survival time revealed no significant effects for either GFAP or Sparc protein level (Figure 4(b)). In addition, the Kaplan-Meier survival analyses also showed no significant survival time influencing effects for the protein data of either GFAP or Sparc (Figure 4(c)). But interestingly, for GFAP protein expression a significant prognostic association was found in multivariable regression analyses with adjustment for sex, age at diagnosis, and therapy regimen (*p* = 0.016, hazard ratio: 2.3854, Supplemental table S2H).

### 3.5. Association of mRNA and Protein Data with Age at Diagnosis and Gender of GBM Patients

**Figure 5:**
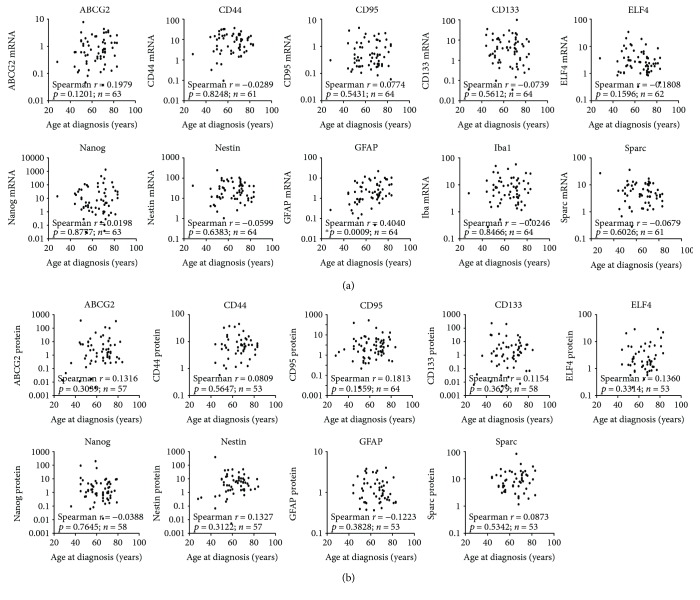
Expression of candidate stem cell and differentiation markers in association with age at diagnosis. (a) Illustration of the correlation analyses between mRNA expression of candidate stem cell marker (ABCG2, CD44, CD95, CD133, ELF4, Nanog, and Nestin) or potential differentiation marker (GFAP, Iba1, and Sparc) and patients' age at diagnosis determined by Spearman's nonparametric correlation in GBM samples. mRNA was analyzed by quantitative RT-PCR with normalization to 18S rRNA, ^∗^ *p* < 0.05. (b) Illustration of the correlation analyses between protein expression of candidate stem cell marker (ABCG2, CD44, CD95, CD133, ELF4, Nanog, and Nestin) or potential differentiation marker (GFAP, Iba1, and Sparc) and patients' age at diagnosis determined by Spearman's nonparametric correlation in GBM samples. Protein expression was determined by immunoblot analyses with normalization to GAPDH, no significant associations.

Since age at diagnosis is an important prognostic factor for GBM patients [23], we next investigated whether a correlation between age at diagnosis and candidate marker expression exists. At the level of mRNA, we found a significant correlation only for GFAP (Figure 5(a)). Higher age at diagnosis was associated with an increasing GFAP mRNA expression (Spearman *r*: 0.4040, *p* = 0.0009). In contrast, at the protein level (Figure 5(b)), we found no significant correlation between age at diagnosis and any of the analyzed stem cell markers (ABCG2, CD44, CD95, CD133, ELF4, Nanog, or Nestin) or the differentiation markers (GFAP and Sparc).

**Figure 6:**
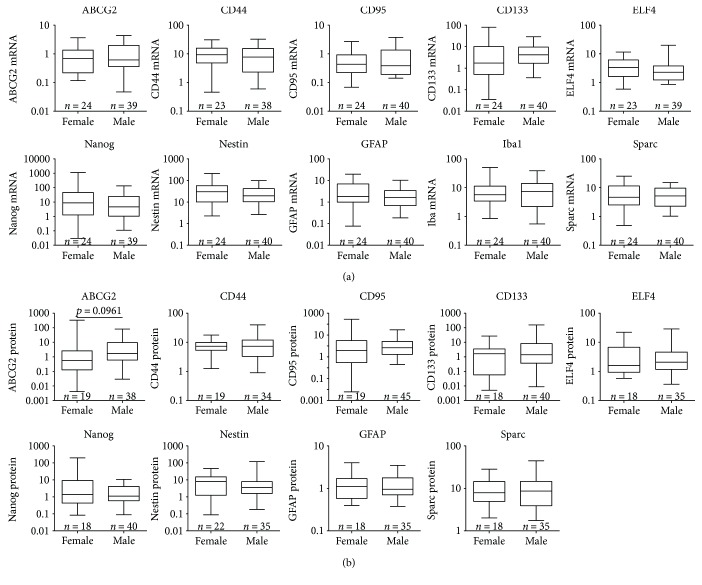
Association analysis of expression of candidate stem cell and differentiation markers with the gender of the GBM patients. (a) Illustration of the association analyses between mRNA expression of candidate stem cell marker (ABCG2, CD44, CD95, CD133, ELF4, Nanog, and Nestin) or potential differentiation marker (GFAP, Iba1, and Sparc) and the gender of GBM patients. Data are shown as box plots representing the median as horizontal bars as well as the 5th and 95th percentiles. mRNA level was analyzed by quantitative RT-PCR with normalization to 18S rRNA, Mann–Whitney *U* test, no significant differences. (b) Illustration of the association analyses between protein expression of candidate stem cell marker (ABCG2, CD44, CD95, CD133, ELF4, Nanog, and Nestin) or potential differentiation marker (GFAP, Iba1, and Sparc) and the gender of GBM patients. Data are shown as box plots representing the median as horizontal bars as well as the 5th and 95th percentiles. Protein expression was determined by immunoblot analyses with normalization to GAPDH, Mann–Whitney *U* test, no significant associations.

Concerning patient gender, mRNA and protein expression of the candidate genes were not significantly different in GBM tissue between female and male patients (Figures 6(a) and 6(b)). Only for ABCG2 protein expression there was a trend towards a higher ABCG2 level in male GBM patients (*p* = 0.0961).

### 3.6. Expression of Candidate Stem Cell as well as Differentiation and Microglia Markers in Glioma Stem Cell-Like LN18 Neurospheres

Additionally, we investigated the expression of stem cell, differentiation, and microglia markers in stem cell-like LN18 neurospheres in comparison to the adherent counterpart as well as to U87 MG, another GBM cell line widely used. Further, the pluripotent embryonal carcinoma cell line NTERA-2 was used as a positive control for immunoblot analyses since these cells display characteristics of human neuronal progenitor cells and show expression of the stemness marker Nestin [24].

**Figure 7:**
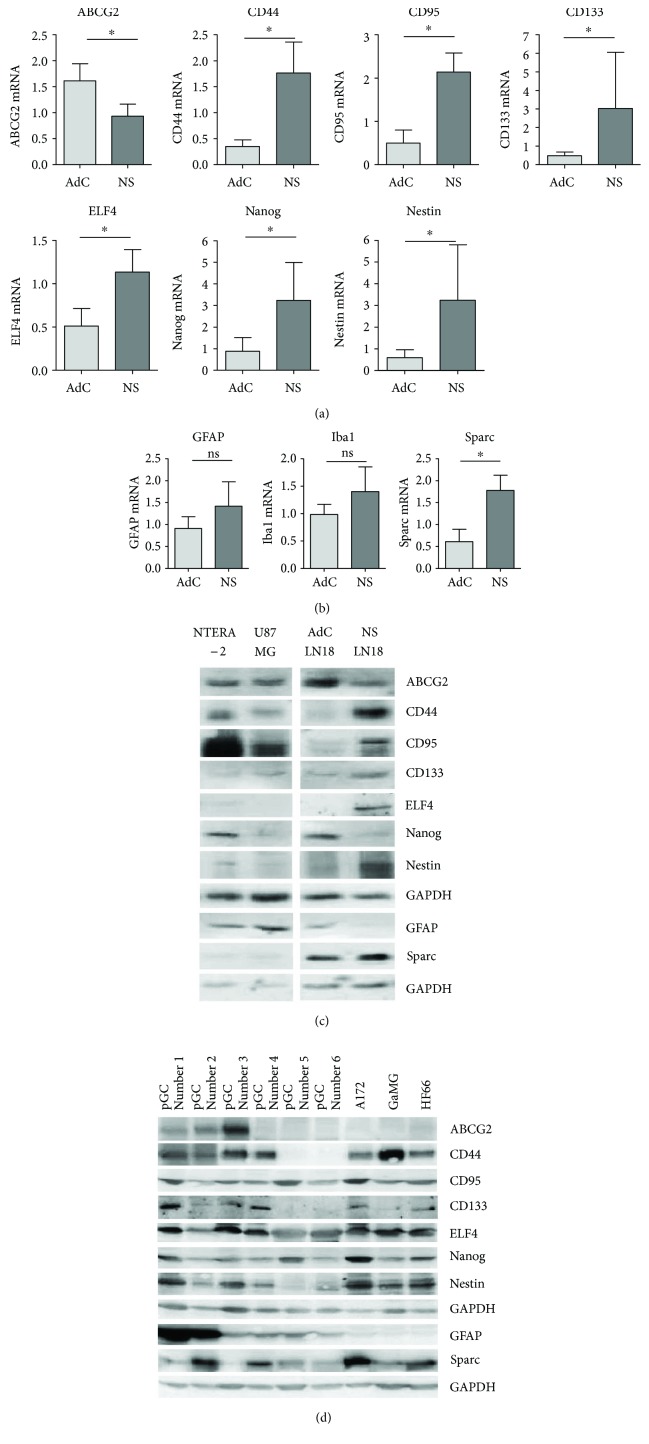
Expression of potential stem cell proteins as well as differentiation markers in adherent GBM cells and stem cell-like neurospheres. (a) Relative mRNA expression of the candidate stem cell markers ABCG2, CD44, CD95, CD133, ELF4, Nanog, and Nestin in adherent LN18 cells and stem cell-like neurospheres analyzed by quantitative RT-PCR with normalization to 18S rRNA and shown as mean with standard deviation of four independent experiments. Mann–Whitney *U* test, ^∗^ *p* < 0.05. (b) Relative mRNA expression of the differentiation marker GFAP as well as the microglia markers Iba1 and Sparc in adherent LN18 cells and stem cell-like neurospheres analyzed by quantitative RT-PCR with normalization to 18S rRNA and shown as mean with standard deviation of four independent experiments. Mann–Whitney *U* test, ^∗^ *p* < 0.05; ns = not significant. (c) Protein expression of the candidate stem cell markers (ABCG2, CD44, CD95, CD133, ELF4, Nanog, and Nestin) and the differentiation marker GFAP as well as the microglia marker Sparc in adherent LN18 cells and stem cell-like neurospheres determined by Western blot. GAPDH was used as loading control, a representative immunoblot of 3-4 independent experiments. (d) Protein expression of the candidate stem cell markers (ABCG2, CD44, CD95, CD133, ELF4, Nanog, and Nestin) and the differentiation marker GFAP as well as the microglia marker Sparc in primary GBM cells (pGC) and GBM cell lines (A172, GaMG, and HF66) determined by Western blot. GAPDH was used as loading control.

We found a significant downregulation of ABCG2 mRNA expression (Figure 7(a)) from 1.62 (±0.33) in adherent cells to 0.93 (±0.23) in stem cell-like LN18. This was strikingly contrasted by a significant upregulation of all other investigated stem cell marker mRNA levels in stem cell-like LN18 neurospheres compared to adherent LN18 cells: CD44 from 0.35 (±0.13) to 1.77 (±0.59), CD95 from 0.49 (±0.30) to 2.14 (±0.44), CD133 from 0.48 (±0.19) to 3.03 (±3.02), ELF4 from 0.51 (±0.20) to 1.13 (±0.26), Nanog from 0.88 (±0.63) to 3.24 (±1.75), and Nestin from 0.59 (±0.36) to 3.24 (±2.55).

The differentiation marker GFAP and the microglia marker Iba1 were also expressed at mRNA level in LN18 cells but without any significant alterations between adherent cells and neurospheres (Figure 7(b)). In contrast, the microglia marker Sparc was about threefold upregulated from 0.61 (±0.28) in adherent LN18 cells to 1.78 (±0.35) in stem cell-like neurospheres.

The corresponding protein expression of adherent LN18 cells and their derived neurospheres in comparison to the NTERA-2 pluripotent embryonal carcinoma cells and U87 MG glioma cells is seen in Figure 7(c). In agreement with its decreasing mRNA level, we found a reduced expression of ABCG2 protein in LN18 neurospheres. In contrast to the increased Nanog mRNA level in stem-like neurospheres, we found a decreased Nanog protein content in LN18 neurospheres compared to the corresponding adherent cells. All other investigated candidate stem cell markers (CD44, CD95, CD133, ELF4, and Nestin) were upregulated in LN18 neurospheres in concordance with the mRNA data. These stem cell markers were also found in pluripotent NTERA-2 cells with a particularly high expression of CD95, CD44, ABCG2, and Nanog which is to date only described for Nanog [25]. CD133 and ELF4 were only marginally expressed in NTERA-2 cells, and Nestin was also detectable as already described by Pleasure and Lee [24].

Protein expression of the differentiation marker GFAP was strongly reduced in the stem cell-like LN18 neurospheres compared to the adherent cells but showed also a high expression in NTERA-2. The microglia marker Sparc was expressed higher in LN18 neurospheres compared to adherent LN18U87 MG which showed a higher basal expression of CD95, CD44, and GFAP but lower levels of ABCG2, Nanog, and Sparc than adherent LN18 GBM cells. A nearly comparative protein expression was found for CD133 and Nestin whereas ELF4 was undetectable in both LN18 and U87 MG adherent GBM cells. The microglia marker Iba1 was below the detection limit in all investigated cell types despite using three different antibodies (not shown).

### 3.7. Expression of Candidate Stem Cell and Differentiation Markers in Primary GBM Cells and GBM Cell Lines

Finally, expression of stem cell-associated and differentiation marker proteins was analyzed in primary GBM cells (pGC) isolated from six different fresh tumor samples in comparison to three further GBM cell lines (A172, GaMG, and HF66). As seen in Figure 7(d), all investigated primary GBM cells and GBM cell lines express to a different content both stem cell and differentiation markers which prove the CSC characteristics in the tumor samples and shows how diverse the expression profile might be in GBM cells isolated from different patients. Noteworthy, GBM cells having a high CD133 and Nestin protein expression (e.g., pGC#1, A172) also show particularly high levels of Nanog. Concerning GFAP protein expression, it was remarkable that the primary GBM cells had substantially higher levels than the GBM cell lines (A172, GaMG, and HF66). ABCG2 was detectable in three out of six primary GBM cells and almost missing in the GBM cell lines. A particularly high CD44 expression was seen in the GaMG cell line. In two of the primary GBM cells, CD44 was under the detection limit. Protein expression of CD95, ELF4, and Sparc was highly variable between the different GBM cells.

## 4. Discussion

Glioma stem cells have emerged as a crucial player in human GBM and are thought to be responsible for resistance to conventional therapy resulting in the rapid occurrence of tumor relapses [3]. To date, mRNA and/or protein expression of individual potential glioblastoma stem cell markers has only been analyzed in different and divergent patient cohorts [13, 16–18, 26, 27]. Therefore, we concurrently investigated the expression of the most-accepted GBM stemness proteins CD133 [4, 6] and Nestin [28] as well as other potential stem cell markers, based on several experimental studies, including the efflux transporter ABCG2 [14] and the transmembrane receptors CD44 [13] and CD95 [29] as well as the transcription factors ELF4 [11] and Nanog [12].

The well-characterized Nestin was excessively upregulated in GBM tissues compared to nonmalignant brain at both mRNA and protein level which matches with the investigations from others [26]. Since Nestin is discussed to play a role in cell survival and proliferation of cancer and stem cells [28], one might expect that expression of Nestin in GBM is associated with patients' survival. Such a prognostic influence of Nestin expression in GBM was shown by Wu and colleagues [30]. Our univariate survival curves of Nestin protein data showed a trend towards a better survival time in patients expressing lower Nestin levels. But even more important, multivariable analyses with adjustment for sex, age at diagnosis, and therapy regime revealed a significant association between Nestin protein expression and patients' survival. Zhang and colleagues also found that survival rate was significantly decreased in GBM patients with strong Nestin expression [26], whereas this survival-associated effect could not be confirmed by a study of Kim and colleagues [31].

A close correlation has been observed between CD133 expression and chemoresistance as well as GBM survival [7, 27, 30]. Recent works depict that also CD133-negative glioma stem-like cells exist [32–34] and that the expression of CD133 may reflect the environmental conditions and stress responses [31, 35]. Thus, both an increased and an unchanged expression status of CD133 might exist in GBM depending on the tumor region or on the GBM subtype. In contrast to Kim and colleagues [31], we found a significant association between CD133 expression and the survival time of GBM patients. Patients with a high CD133 protein content had a worse prognosis than those with low CD133 expression. This agrees with the association between CD133 expression and chemoresistance as well as radiation resistance [7, 8]. A targeted therapy against CD133 might therefore represent a promising tool for GBM treatment as discussed by Choy and colleagues [36]. Of note, however, CD133 expression is not specific for GBM stem cells, since it is widely distributed in several stem/progenitor cells as well as tumor cells of other origin, and several models have shown the stem cell capacity of CD133-negative GBM cells [36, 37]. These observations may indeed jeopardize establishing a CD133-dependent targeted therapy specifically directed towards GBM stem cells. If it is possible in the future to directly target only GBM cells, maybe by virus-directed intracellular trafficking of plasmids with CRISPR-Cas9 technology as an important tool for genome editing, it would be conceivable to eradicate tumor stem cells or shift them to a more differentiated cell type being more vulnerable for cancer therapies.

The transcription factor myeloid Elf-1 like factor ELF4 (also known as MEF) was postulated as gatekeeper gene in gliomagenesis and promotes stem cell characteristics [11]. ELF4 is highly expressed in both human and mouse GBM, and GBM patients with low ELF4 expression exhibit better survival times [11]. Our GBM patient cohort showed also a markedly elevated ELF4 expression but we found no significant association with the survival time. One explanation for this difference to the above-mentioned data from Bazzoli and colleagues [11] might be the heterogeneity of the GBM.

A further transcription factor, which was associated with the stem cell phenotype and progression of GBM, is Nanog [12] which has been implicated in the malignancy of GBM by regulating GBM stem cell tumorigenicity, clonogenicity, and proliferation [38, 39]. Correspondingly, we found elevated Nanog expression in our GBM patient cohort. Interestingly, a significant association of Nanog protein level with survival of GBM patients was seen when patients were subdivided in two groups depending on their survival time (≤median versus >median). A similar trend was found in the Kaplan-Meier survival curves: patients having a high Nanog protein content showed a poorer survival time (354.5 days) than patients with a lower Nanog protein level (435 days, HR: 0.6325) but this effect was not seen in multivariable analysis.

Nanog is activated by CD44 [40] which represents the receptor for the glycosaminoglycan hyaluronan [41]. A study of Eibl and colleagues indicated that CD44 variants are expressed in 100% of all GBM cell lines and tumors [42]. Furthermore, CD44 is coexpressed with CD133 in GBM neurospheres [43] and inhibition of CD44 affects progression of GBM in mice [44]. However, despite high expression levels of CD44 in our investigated GBM samples, no association of CD44 expression with patients´ survival time was observed.

Among the investigated candidate stem cell markers, CD95 was the only one which was downregulated at its mRNA level but this did not translate to the protein expression. Nevertheless, we found a significant association of CD95 mRNA with the survival time: GBM patients with a high CD95 expression showed a poorer survival (161 days) than patients with a low CD95 mRNA content (373 days, HR: 0.6582). Overexpression of CD95 is considered to be an important survival factor for tumor cells and is described as potential stem cell marker [29]. Interestingly, a recent phase II study using a CD95L-binding fusion protein in glioblastoma demonstrated that CD95 pathway inhibition in combination with radiotherapy represents an innovative concept with clinical efficacy [45].

However, differentiation of neural stem cells and glial cells results in an upregulation of the astrocytic marker GFAP which is also expressed in glioma cells [28, 46, 47]. Compared to nonmalignant brain, expression of GFAP was nearly the same in the GBM specimens and there was no significant association with the survival time. A further marker for astrocytes and glial cells is the secreted protein acidic and rich in cysteine (Sparc, osteonectin) which is also highly expressed in GBM where it promotes the migratory and invasive behavior of glioma cells [48, 49]. In our patient cohort, Sparc was significantly upregulated but there was no significant association with the survival time. Capper et al. found also no association between the level of Sparc in tumor cells and patient's survival, but increased tumor vascular Sparc expression was associated with decreased patient's survival [50]. In contrast, our work represents a global expression analyses in the whole tumor mass without tumor region-dependent or cell type-specific differentiation which might be in fact a study limitation, particularly when considering the heterogeneity of GBM between different patients. It has to critically take into account that different types of GBM have been discovered by the identification of distinct genetic and epigenetic profiles [51]. Thus, it seems possible that predictive molecular biomarkers for therapy response and outcome are different in the respective GBM subtypes. Our patient cohort was not classified, and therefore, we can draw no conclusion regarding such plausible differences in GBM subtypes. Further, it has to be noted that within the same tumor hypoxic, necrotic and vital areas exist which certainly have different types of expression profiles. In our study, only vital tumor samples from the edge region were used for expression analyses to reduce the influence of, for example, hypoxia and necrosis to a minimum.

The GSC growth seems to depend on the brain microenvironment, and nontumorigenic cell types such as microglia and endothelial cells might be able to influence GSC phenotype transition from precursor to differentiated cells and vice versa [9, 10]. It has been shown that microglia can stimulate the invasiveness of glioma cells [21] and local depletion of microglia and macrophages from glioma has been shown to significantly reduce tumor burden and prolong life expectancy in mice [52]. Therefore, we analyzed the expression of Iba1, which is specifically expressed in microglia but not in other cell types of the brain [53]. Unfortunately, the Iba1 protein was not detectable in our samples. This may be due to low expression levels; however—despite using several antibodies—we also cannot exclude a technical reason. Iba1 mRNA content, however, was significantly increased in GBM specimens in comparison to nonmalignant brain but there was no association with patients' survival time.

Cultured GBM cells have the ability to form stem-like neurospheres which were capable of *in vivo* tumor formation when injected into nude mice, whereas non-sphere-forming cells isolated from GBM did not grow as tumor [6]. Brescia et al. reported that disruption of CD133 expression in human GBM neurospheres impaired the self-renewal and tumorigenic capacity of neurosphere cells [54] demonstrating that this cell model is suitable for investigating the stem cell behavior of GSCs. In accordance with the work of Yuan and colleagues [6], we found an elevated expression of both CD133 and Nestin in LN18 neurospheres. Except for ABCG2, which was downregulated, CD44, CD95, ELF4, and Nanog were markedly elevated in stem-like LN18 neurospheres in comparison to the adherent cells. To date, only one study indicates a role of ELF4 in regulating neurosphere formation [11] while expression and function of both Nanog and CD44 are well-described in GSC neurospheres [12, 55, 56].

ABCG2 (ATP-binding cassette transporter subfamily G member 2), also called BCRP (breast cancer resistance protein), regulates self-renewal and stem cell marker expression but not tumorigenicity or radiation resistance of glioma cells [57]. A study from Warrier and colleagues demonstrated that chemoresistant cancer stem-like neurosphere cells from the human glioblastoma cell line U138MG have an increased mRNA expression of ABCG2 [58] which is not in accordance with our reduced expression of ABCG2 found in stem-like neurospheres of LN18 cells. Differences between our investigation and the study of Warrier et al. are the use of different GBM cell lines and other techniques for measurement of ABCG2 expression which both could influence the results.

Expression of the astrocyte differentiation marker GFAP was strongly downregulated in our stem-like LN18 neurospheres arguing for a decreased differentiation or dedifferentiation. This is in accordance with the upregulation of Nestin since it was shown that an increasing differentiation of neural stem cells and glial cells results in a reduced expression of Nestin combined with an upregulation of GFAP [28, 46]. Whereas Iba1 was unchanged on mRNA and undetectable on protein level, the astrocytic and glial cell marker Sparc was significantly upregulated in stem-like LN18 neurospheres as already seen in our GBM tissue analysis. To our knowledge, we are the first to detect Sparc expression in stem-like neurosphere cells. Thus, further studies concerning the role of Sparc in the pathogenesis of GSCs are urgently needed.

## 5. Conclusions

In summary, we found that several potential stem cell and differentiation markers are dysregulated in stem-like GBM cells as well as in total GBM tissue compared to nonmalignant brain. For most of these markers, the prognostic potential appears to be limited since their expression levels did not correlate with patients' survival. Only the expression of CD133 and Nestin was considerably associated with the survival time of GBM patients and was also markedly upregulated in stem-like neurospheres representing potential candidates for targeted GBM therapy. Nevertheless, the investigated stem cell markers could have tumor-promoting effects regardless of any prognostic association. Also, several other proteins are discussed to be potential stem cell markers and should be intensively studied in further investigations to identify perspective prognostic and therapeutic targets.

## Figures and Tables

**Table 1:** Clinicopathological characteristics of tumor specimens.

Characteristic	Value
*N* (primary tumor)	77
Median age at diagnosis	67
(25th percentile; 75th percentile)	(54.5;72)
Age classes, *N* (%)	
<50 years	6 (7.8)
50 to <60 years	23 (29.9)
60 to <70 years	17 (22.1)
70 to <80 years	26 (33.8)
>80 years	5 (6.5)
Gender, *N* (%)	
Male	50 (64.9)
Female	27 (35.1)
Male-to-female ratio	1.9
Vital status at study end, *N* (%)	
Dead	68 (88.3)
Alive	9 (11.7)
Resection grade, *N* (%)	
Total	45 (58.4)
Subtotal	32 (41.6)
Therapy regimen^1^, *N* (%)	
Radiotherapy and chemotherapy	45 (58.4)
Only radiotherapy	13 (16.9)
Other regimens	4 (5.2)
No adjuvant therapy	9 (11.7)

^1^Six therapy regimens could not be assessed.

## Supplementary Materials

Supplementary Materials Supplemental Tables S1A–J: Multivariable Cox regression analysis of mRNA data of the investigated stem cell as well as differentiation markers for patients with GBM. Data were adjusted for sex, age at diagnosis, and therapy regime (RCT: radiochemotherapy; RT: only radiotherapy; no T: no adjuvant therapy). Supplemental Tables S2A–I: Multivariable Cox regression analysis of protein data of the investigated stem cell as well as differentiation markers for patients with GBM. Data were adjusted for sex, age at diagnosis, and therapy regime (RCT: radiochemotherapy; RT: only radiotherapy; no T: no adjuvant therapy).
