# Supplementary material for: Association of Glioblastoma Multiforme Stem Cell Characteristics, Differentiation, and Microglia Marker Genes with Patient Survival
Source: Stem Cells Int. 2018 Jan 17;2018:9628289. doi: 10.1155/2018/9628289 (PMC5822829; doi:10.1155/2018/9628289)
Supplement: Supplementary Materials — Supplemental Tables S1A–J: Multivariable Cox regression analysis of mRNA data of the investigated stem cell as well as differentiation markers for patients with GBM. Data were adjusted for sex, age at diagnosis, and therapy regime (RCT: radiochemotherapy; RT: only radiotherapy; no T: no adjuvant therapy). Supplemental Tables S2A–I: Multivariable Cox regression analysis of protein data of the investigated stem cell as well as differentiation markers for patients with GBM. Data were adjusted for sex, age at diagnosis, and therapy regime (RCT: radiochemotherapy; RT: only radiotherapy; no T: no adjuvant therapy). [file 9628289.f1.docx]

Supplemental Tables S1A-J. Multivariable Cox regression analysis of mRNA data of the investigated stem cell as well as differentiation markers for patients with GBM. Data were adjusted for sex, age at diagnosis and therapy regime (RCT, radiochemotherapy; RT, only radiotherapy; no T, no adjuvant therapy).
Supplemental Tables S2A-I. Multivariable Cox regression analysis of protein data of the investigated stem cell as well as differentiation markers for patients with GBM. Data were adjusted for sex, age at diagnosis and therapy regime (RCT, radiochemotherapy; RT, only radiotherapy; no T, no adjuvant therapy).
